# A foundational approach to culture and analyze malnourished organoids

**DOI:** 10.1080/19490976.2023.2248713

**Published:** 2023-09-19

**Authors:** Meryl Perlman, Stefania Senger, Smriti Verma, James Carey, Christina S. Faherty

**Affiliations:** a Mucosal Immunology and Biology Research Center, Division of Pediatric Gastroenterology and Nutrition, Massachusetts General Hospital, Boston, MA, USA; b Department of Pediatrics, Harvard Medical School, Boston, MA, USA; c Division of Infectious Diseases, Massachusetts General Hospital, Boston, MA, USA

**Keywords:** alkaline phosphatase, gastrointestinal, environmental enteric dysfunction, interleukin-8, malnutrition, M cells, mucus, organoids, Shigella, transepithelial electrical resistance

## Abstract

The gastrointestinal (GI) epithelium plays a major role in nutrient absorption, barrier formation, and innate immunity. The development of organoid-based methodology has significantly impacted the study of the GI epithelium, particularly in the fields of mucosal biology, immunity, and host-microbe interactions. Various effects on the GI epithelium, such as genetics and nutrition, impact patients and alter disease states. Thus, incorporating these effects into organoid-based models will facilitate a better understanding of disease progression and offer opportunities to evaluate therapeutic candidates. One condition that has a significant effect on the GI epithelium is malnutrition, and studying the mechanistic impacts of malnutrition would enhance our understanding of several pathologies. Therefore, the goal of this study was to begin to develop methodology to generate viable malnourished organoids with accessible techniques and resources that can be used for a wide array of mechanistic studies. By selectively limiting distinct macronutrient components of organoid media, we were able to successfully culture and evaluate malnourished organoids. Genetic and protein-based analyses were used to validate the approach and confirm the presence of known biomarkers of malnutrition. Additionally, as proof-of-concept, we utilized malnourished organoid-derived monolayers to evaluate the effect of malnourishment on barrier formation and the ability of the bacterial pathogen *Shigella flexneri* to infect the GI epithelium. This work serves as the basis for new and exciting techniques to alter the nutritional state of organoids and investigate the related impacts on the GI epithelium.

## Introduction

Approximately 3.1 million children die from undernutrition each year, and hunger contributes to more than half of global childhood deaths.^1,2^ Undernutrition, which can be caused by micronutrient or macronutrient deficiencies, makes children more vulnerable to illness, exacerbates environmental enteric dysfunction (EED; a chronic inflammatory condition of the intestine prevalent in developing countries), leads to poor surgical outcomes, and may contribute to vaccine failure.^3–6^ Specifically, undernutrition is thought to lead to worse outcomes in diarrheal disease, which is a major cause of childhood mortality throughout the globe.^5,6^ Children with undernutrition experience worse diarrheal illness and are less likely to develop a response or effective immunity to oral vaccines.^5–7^ This combination of factors raises the question of the relationship between undernutrition and the host response to enteric pathogens.

Research has identified the systemic effects of undernutrition and EED in patients, including impaired gut-barrier function, reduced exocrine secretion of protective substances, low levels of plasma complement, lymphatic tissue atrophy, and reduced type IV (delayed) hypersensitivity reactions.^8–10^ However, the effect of malnutrition specifically on the function of the gastrointestinal (GI) epithelium has not been adequately studied.^11^ Understanding mechanistic impacts of malnutrition on the intestinal epithelium would be particularly helpful in improving our understanding of infection and developing preventive and therapeutic interventions for enteric infections, especially for vaccines for use in lower- and middle-income countries (LMICs). Additionally, knowledge of how undernutrition influences the intestinal epithelium would have further applications for patients with other gastrointestinal pathologies, such as intestinal failure (a condition where individuals do not adequately absorb nutrients following intestinal resections or due to malfunction), Crohn’s disease (a form of inflammatory bowel disease (IBD) that can affect any area of the GI tract, often requiring intestinal resection), and in critical illnesses where the lack of enteral nutrition is known to be a factor leading to sepsis.^12–14^ Furthermore, intestinal pathologies requiring surgical resection have more complications in patients with a history of compromised nutrition, as in Crohn’s disease,^15,16^ but a mechanistic understanding into these adverse outcomes is lacking.

The intact epithelium plays a major role in innate immunity and contributes to the activation of the adaptive immune response. The GI epithelium has a particularly important role in sampling antigens directly from the environment. These antigens, like food, pathogens, medications, and allergens, ingested through the mouth are either tolerated or an appropriate immune response is generated in healthy individuals.^17^ Despite this intimate connection with the environment, access to epithelial samples is difficult. Acquiring tissue from patients involves an invasive endoscopic procedure that is ethically and logistically challenging, particularly in children. Furthermore, since severe undernutrition is most prevalent in LMICs, these challenges are heightened. Therefore, much of what we know about the function of the intestinal epithelium in the undernourished state comes from older histological studies,^11,18–23^ which were restricted by the technology available at the time or animal models that have limited applicability to humans.^11^ Understanding the function of the GI tract is further complicated by the fact that the epithelium and the associated immunological sensors are exposed to various foods, microbes, and other variable environmental exposures multiple times a day; and thus, epithelial stimuli, and presumably the associated response, are in constant flux. Therefore, much effort has been devoted to advances in both *in vivo* and *in vitro* modeling of the intestine for research purposes.^24^ For example, sophisticated murine models have been humanized and/or personalized by transferring human immune cells and/or microbiome specimens to better study the GI environment.^25^ These models have considerations including cost, ethical challenges, and applicability to humans. Thus, *in vitro* human modeling of the GI tract has been actively pursued to facilitate research.^24,26–28^ For years cell lines derived from GI cancers such as Caco-2 and HT-29 were used to study epithelial interactions with antigens; but many drawbacks exist, including the fact that cell lines are typically derived from genetically-altered cancer cells of limited complexity and physiological relevance.^29,30^ The development of organoids has evolved to provide more sophisticated and personalized human and patient platforms for studying the GI epithelium.^31^ Intestinal organoids are derived from stem cells present in human tissue, which are accessed during routine endoscopy or colonoscopy procedures or from resected tissue.^28,32^ Once in culture, the organoids develop into three-dimensional (3D) structures that mimic the *in vivo* architecture and cellular differentiation of the tissue of origin.^33^ Furthermore, these 3D structures can be dissociated and seeded onto two-dimensional (2D) transwell systems to facilitate studies with enteric pathogens.^34^

Given the multiple benefits of organoids and organoid-derived monolayers, coupled with the difficulty of accessing tissue from EED patients, we sought to create a malnourished model of the GI tract to study the impacts of macronutrient deficiency on the function of the epithelium. While the morphological appearance and histology of the undernourished GI epithelium has been well documented,^18–21^ little information is available on the mechanistic and metabolic changes that occur during undernutrition. In fact, it has been noted that this paucity of information, particularly with regards to the morphological and functional changes that occur in malnourished children, is a key barrier to understanding malnutrition and developing effective intervention strategies.^11^ We saw organoids as ideal, relatively accessible models to study the effects of nutrient deprivation on the GI epithelium; and thus, began using organoids and organoid-derived monolayers from duodenal samples as proof-of-concept analyses to demonstrate the ability to create malnourished models with widely available techniques, reagents, and tools. Following successful reduction or removal of certain media additives, we established the viability of malnourished models, evaluated gene and protein expression for known markers of malnutrition, and evaluated infection of malnourished monolayers with the enteric bacterial pathogen *Shigella flexneri*.^34,35^ Herein, we describe the methodology and results of our approach and analyses to successfully develop human malnourished organoid models for use in the laboratory setting.

## Results

### Culturing and assessment of the malnourished organoids

**Figure 1.**
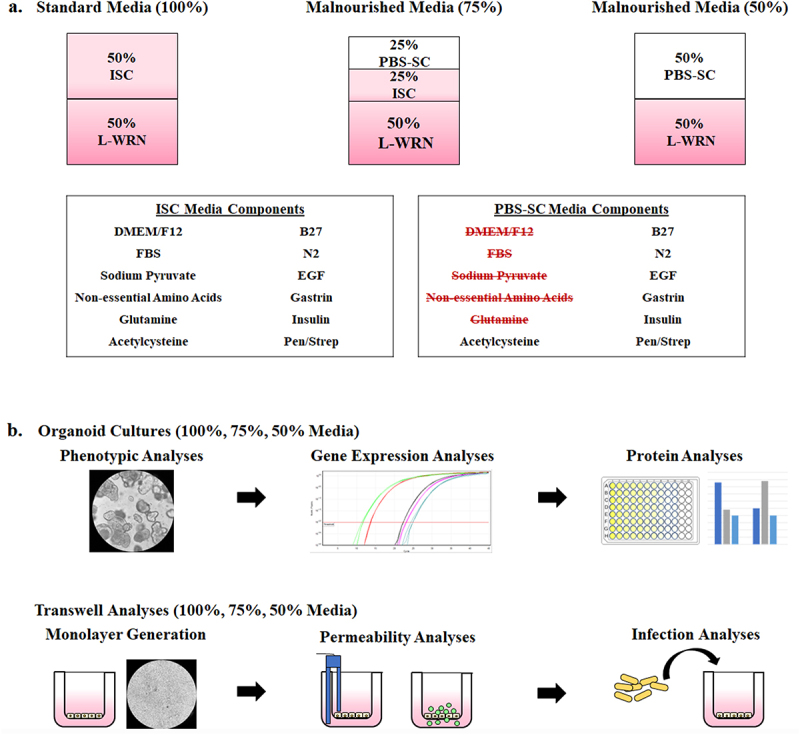
**Malnourished media composition and study design.** To generate malnourished organoids, certain media additives were eliminated to reduce nutritional content while maintaining cellular viability. Following establishment of media formulations to reduce protein, amino acid, and carbohydrate concentrations, both organoids and organoid-derived monolayers were incubated in the various media compositions and subsequently analyzed for the effects of malnourishment.

To first confirm nutrient deprivation was not lethal to host cells, we performed trial experiments with the colonic cell line HT-29. Culture media were diluted with 1X PBS to 50%, 25%, and 10% of the standard 100% media formulation, and the cells were incubated and monitored over the course of six days. With the exception of the 10% formulation that served as a negative control for cellular viability, the 50% and 25% formulations preserved cellular viability and similar cellular morphological features, especially the 50% formulation (data not shown). Knowing it was possible to preserve viability despite nutrient reduction, we applied a similar technique to human duodenal organoids. After some trial and error, we confirmed that reductions in the L-WRN conditioned medium, which contains critical factors Wnt3A, R-spondin 3 and Noggin that sustain proliferation of intestinal stem cells,^36^ prevented successful generation of organoids (data not shown). Thus, we ultimately developed the 75% and 50% media formulations (Figure 1). We removed either half (75%) or all (50%) of the DMEM/F12 medium, fetal bovine serum (FBS), sodium pyruvate, non-essential amino acids, and glutamine from the intestinal stem cell (ISC) media. For each component that was removed, the equivalent volume was replaced with 1X PBS, thus generating the PBS-SC media. The 75% media formulation used a 1:1 ratio of ISC and PBS-SC, while the 50% media formulation used PBS-SC in place of ISC. Both the 75% and 50% media formulations maintained the standard L-WRN composition and concentration to ensure cellular viability (Figure 1).

**a.** **Malnourished media.** The 50% L-WRN was essential to cellular viability and could not be removed or diluted. However, certain components of the ISC media could be removed without detrimental effects to cellular viability, and the volume was replaced with 1X PBS to generate 50% PBS-SC media. The standard media formulation (100%) is composed to 50% ISC media and 50% L-WRN media. The 75% malnourished media is composed of 25% PBS-SC, 25% ISC, and 50% L-WRN. The 50% media formulation is composed of 50% PBS-SC and 50% L-WRN. Overall, by diluting the ISC media, the concentration of protein, amino acids, and carbohydrates was reduced. All media types (100%, 75%, and 50%) also contained the inhibitors Y-27632 and A 83-01 in the L-WRN portion of the media.

**b.** **Malnourished organoid and organoid-derived monolayer analyses.**

**Top:** Organoids from three separate donors were cultured in the three different media formulations for seven days. The organoids were then analyzed for changes in phenotypic appearance, gene expression via qRT-PCR, and protein expression via immunofluorescence analysis and lactate dehydrogenase release, intestinal alkaline phosphatase secretion, and interleukin-8 secretion assays.

**Bottom:** The media formulations were also used to culture malnourished organoid-derived monolayers (mHIODEM). Following organoid generation in 100% media for seven days, organoids from the three separate donors were trypsinized, the cells were seeded onto transwells, and the monolayers were cultured for 13 days in the three different media formulations. The monolayers were differentiated with DAPT for the last 2 days of culturing (starting on day 11). Afterwards, the monolayers were analyzed for permeability via TEER and FITC-dextran passage, as well as susceptibility to infection via *S.* *flexneri* invasion analyses.

**Figure 2.**
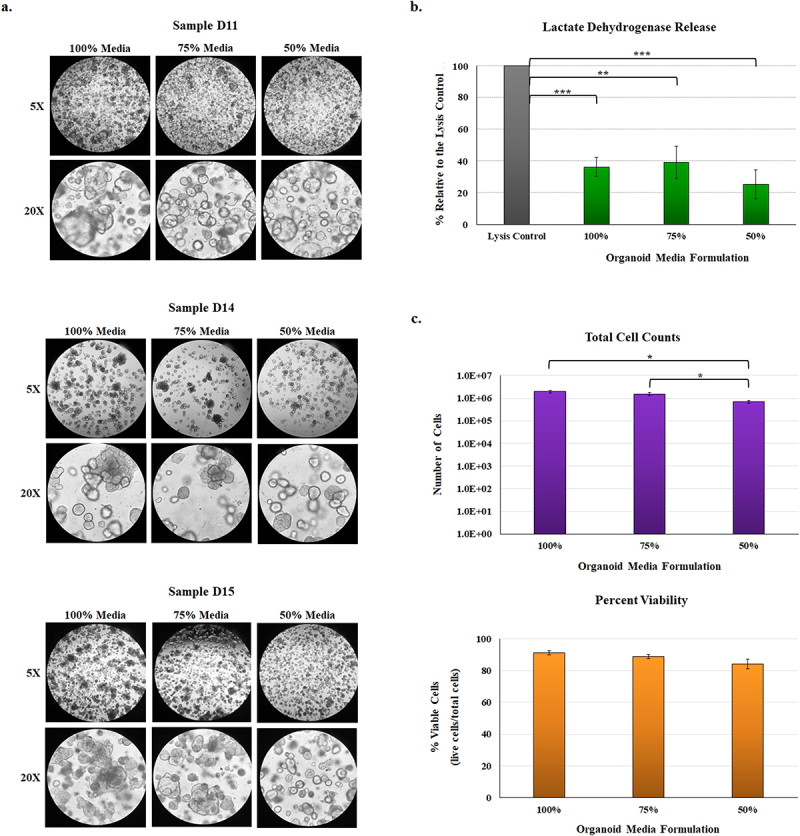
Phenotypic analysis of malnourished organoids.

Three different duodenal organoid lines (D11, D14, and D15) derived from separate patients cultured in the 75% and 50% media formulations retained the same morphological features as the 100% media formulation (Figure 2a). The 50% formulation at times produced more discrete and morphologically less-differentiated organoids, but the viability was preserved and enabled us to proceed with our analyses on day seven of culturing. Additionally, the phenotypes across the D11, D14, and D15 samples were consistent for each of the media formulations (Figure 2a), demonstrating biological reproducibility of the approach. The release of lactate dehydrogenase (LDH), which occurs during host cell death,^37^ was used to measure cellular viability of the malnourished organoids. The amount of LDH in the supernatants of organoids from all media formulations collected on day seven of culturing was significantly reduced relative to the positive lysis control, and there were no significant differences between the media formulations (Figure 2b). To further confirm the viability of the malnourished organoids, we cultured D14 organoids in the three media formulations, trypsinized the cells, and performed cell counts with an automated cell counter following trypan blue staining of the cells. The total cell counts (both live and dead cells) for the 100% and 75% media formulations averaged between 1.0 to 2.5 × 10^6^ cells/ml while the 50% media formulation averaged 5.2 to 8.7 × 10^5^ cells/ml, which was statistically lower (*p* < .05) compared to both the 100% and 75% media formulations (Figure 2c). Despite the lower cell numbers in the 50% media formulation, the percent viability, which was calculated as the number of live cells divided by the total number of cells, averaged between 84% to 91% for all three media formulations (Figure 2c). The data indicate that while the 50% media resulted in slightly slower cell growth, the viability remained the same relative to the 100% and 75% media formulations. Overall, the data support the morphological assessment and demonstrate that our approach to the malnourished media composition and organoid culturing did not result in significant cellular death.

**a.** **Phenotypic evaluation.** Following culturing in the three media formulations, organoids derived from three separate donor samples (D11, D14, and D15) were evaluated phenotypically for changes in appearance. Overall and despite the reduced nutrient concentrations in the malnourished media, organoids matured and developed the characteristic sphere shape. Images above were examined at 5X (top) and 20X (bottom) magnifications for all three sets of samples. The images represent three independent experiments, each with two technical duplicates, for the three organoid lines. The data indicate that organoid development in malnourished conditions is consistent across the three separate donor samples.

**b.** **Lactate dehydrogenase (LDH) release.** Culture supernatants were collected from D11, D14, and D15 organoids following seven days of culture in the 100%, 75%, or 50% media formulations. The data represent the average of four biologically independent experiments from all three lines ± the standard errors of the mean (SEM), and are plotted relative to the positive lysis control provided by the kit. All samples had significantly less LDH release relative to the positive control (**, *p* <.01; ***, *p* <.001). There were no significant differences in LDH release across the organoid media formulations.

**c.** **To examine cellular viability**, D14 organoids were cultured in the three media formulations for seven days. Subsequently, the organoids were trypsinized and stained with trypan blue to obtain total cell numbers and the percent viability of the cells, calculated as the percent of live cells relative to the total number of cells. The 50% media formulation had reduced total cell counts (*, *p* <.05) compared to the 100% and 75% media formulations. However, there were no significant differences in the percent viability across the treatments. For both analyses, data represent the average of three biologically independent experiments ± the SEM, and each experiment had three technical replicates.

### Gene expression analyses in the malnourished organoids

**Figure 3.**
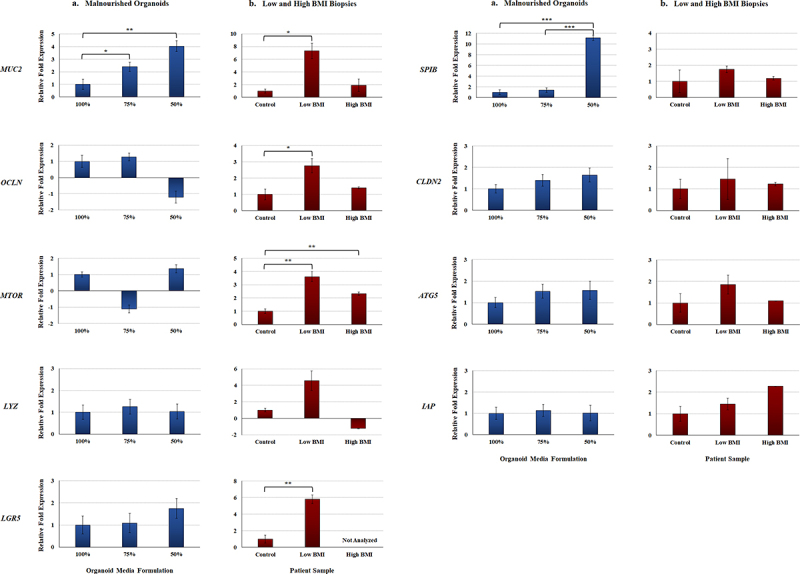
**Gene expression analyses** of malnourished organoids and patient biopsy samples with different BMIs. Quantitative RT-PCR was performed for the indicated genes. Data bars represent the average fold change ± the SEM. All data were normalized to the expression of the 18S housekeeping gene for each experiment. Statistical significance was determined with the Student’s T-test of the ΔCT and the treatment comparisons that are statistically different are indicated for each gene and comparison. Data were considered significant at a *p* value of < .05 (*, <.05; **, <.01; ***, <.001). Please note the different y-axes for each graph.

All gene expression analyses were performed on RNA extracted from organoids cultured for seven days. We also analyzed gene expression from duodenal tissue isolated from biopsies of three clinically undernourished pediatric patients with low body mass index (BMI) scores, three age- and gender-matched controls (healthy BMI), and one obese child (high BMI) to determine if the gene expression patterns from the malnourished organoids reflected a clinical undernourished state. The most interesting result was the expression of the *MUC2* gene for mucin produced by goblet cells^38^, which was significantly increased in the malnourished organoids at both 75% and 50% media formulations as well as in the low BMI biopsy samples (Figure 3). Additionally, *SPIB*, a marker that identifies the specialized M cell population involved in antigen-sampling and is an access point for *S. flexneri* invasion,^34,39^ was significantly upregulated in the 50% media formulation relative to both the 100% and 75% media formulations. Expression of *SPIB* was nearly doubled in the low BMI samples relative to the control, but the increase was not statistically significant. For tight junction and barrier formation markers, the *OCLN* occludin gene of the tight junction complex^40^ was significantly induced in the low BMI biopsies but remained statistically unchanged in the malnourished organoids. The expression of the *CLDN2* claudin-2 gene that encodes a pore forming protein in the tight junction complex^40–43^ trended upwards in both malnourished organoids but was not significant. The autophagy genes mammalian target of rapamycin (*MTOR*)^44^ and autophagy related protein 5 (*ATG5*)^45^ displayed interesting gene expression patterns. *MTOR* displayed potential differential expression in the malnourished organoids but the changes were not statistically significant; however, *MTOR* was significantly induced in both the low BMI and high BMI biopsy samples. *ATG5* had a more consistent expression patterns that trended toward induced expression in both malnourished organoids and the low BMI samples, but the differences were not significant. Two genes, lysozyme (*LYZ*) produced by Paneth cells^46,47^ and intestinal alkaline phosphatase (*IAP*),^48^ remained at constant expression levels in the malnourished organoids, but showed increased, although not significant, expression profiles in the low BMI biopsy samples (Figure 3). Finally, the stem cell marker *LGR5* ^46^ trended toward induced expression in the malnourished organoids and was significantly induced in the low BMI patient biopsies.

**a.** **Malnourished organoids.** Data are expressed as the average fold change relative to the 100% media formulation. Data for each gene represent expression values from the D11, D14, and D15 organoid lines, each cultured in the 100%, 75%, and 50% media formulations. A total of three biologically independent experiments were performed, and each experiment had technical duplicates.

**b.** **Biopsy samples from low and high BMI individuals.** Data are expressed as the average fold change relative to the healthy control biopsy samples. Gene expression data for both the healthy control and low BMI samples were collected from three independent donors (biological replicates), while the high BMI samples was from one donor. Each experiment had technical duplicates.

### Protein expression analyses in the malnourished organoids

**Figure 4.**
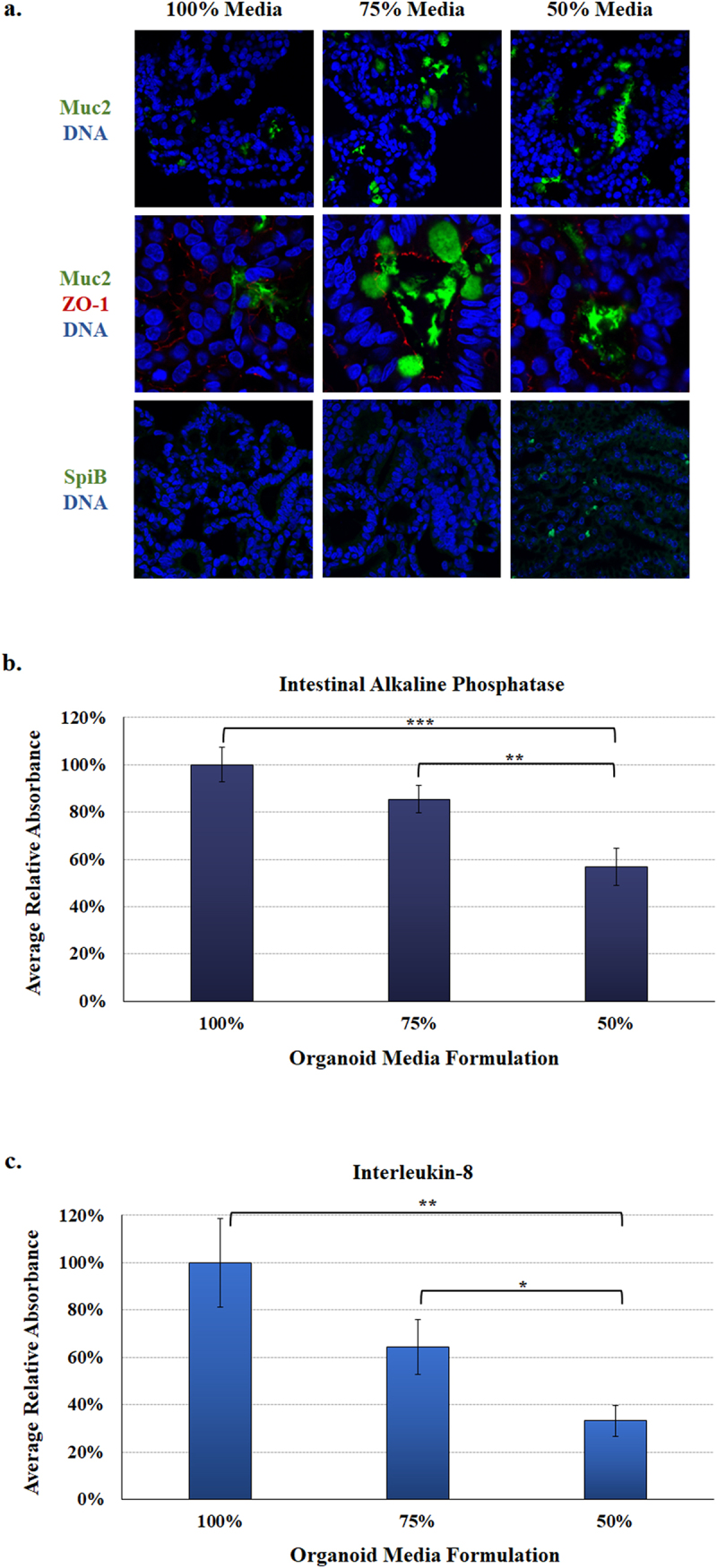
**Protein expression in the malnourished organoids.** Analyses were performed as a measure of protein expression in the organoids with the various media formulations.

To verify the gene expression analyses, immunofluorescence analysis was performed on D14 organoids cultured for seven days in the three media formulations and processed for confocal immunofluorescence analysis (Figure 4a). Analyses for Muc2 expression confirmed induced expression in organoids cultured in both the 75% and 50% media formulations. The Muc2 analyses were performed with and without staining for the tight junction protein zona occludens-1 (ZO-1) to help differentiate between mucus localized inside goblet cells or secreted into the apical center of the spheroid. We also examined SpiB expression in the organoids and found that organoids cultured in the 50% media formulation had induced expression relative to organoids cultured in the 100% and 75% media formulations. Both Muc2 and SpiB analyses confirmed the gene expression data.

**a.** **Immunofluorescence analysis.** D14 organoids were cultured for seven days in the 100%, 75%, or 50% media formulations and subsequently processed and stained for microscopy. The colors of each antibody or DNA stain (blue) are indicated. Muc2 (green) expression, which was analyzed with and without ZO-1 (red) staining to help differentiate secreted Muc2 versus Muc2 localized to goblet cells, was induced in the 75% and 50% media formulations. SpiB (green) expression was induced in the 50% media formulation. The immunofluorescence confirms the gene expression analyses. Please note the Muc2 and ZO-1 images are magnified so the red ZO-1 signal can be seen. All images were captured with a 60X objective.

**b.** **Intestinal alkaline phosphatase (IAP).** Culture supernatants were collected after seven days of culture from the D11, D14, and D15 organoid lines and measured in four biologically independent experiments. The average absorbance reading ± the SEM for each media formulation is plotted relative to the 100% control media. Statistical significance was determined by a Student’s T-test, and data were considered significant at a *p* value of < .05 (*, <.05; **, <.01; ***, <.001). There was a significant decrease between the 100% and 50% media formulations, as well as the 75% and 50% media. There was no significant difference between the 100% and 75% media.

**c.** **Interleukin-8 (IL-8) secretion.** Culture supernatants were analyzed from biological and technical replicates as noted above, with the same statistical analyses and significance considerations. There was a significant difference between the 100% and 50% media, as well as the 75% and 50% media formulation. There was no significant difference between the 100% and 75% media formulation.

To further examine protein expression in the malnourished organoids, culture supernatants from the organoids were collected on day seven to examine two markers of malnutrition. First, decreased IAP protein expression has been reported in samples analyzed from the proximal small intestine of malnourished patients.^49,50^ We detected significantly less IAP in both malnourished organoids, with the greatest reduction in the organoids cultured in the 50% media formulation (Figure 4b). Second, interleukin-8 (IL-8) secretion is an important epithelial response to enteric pathogens,^51,52^ and is decreased in malnourished individuals.^53^ Relative to the 100% media control, IL-8 expression in the malnourished organoids trended toward decreased expression in the 75% media formulation while organoids cultured in the 50% media formulation were statistically decreased at approximately 35% of control levels (Figure 4c). The combined IAP and IL-8 analyses indicate that malnourished organoids reflect the expected response of malnourished intestinal epithelium.

### Establishment and analysis of malnourished organoid-derived monolayers

**Figure 5.**
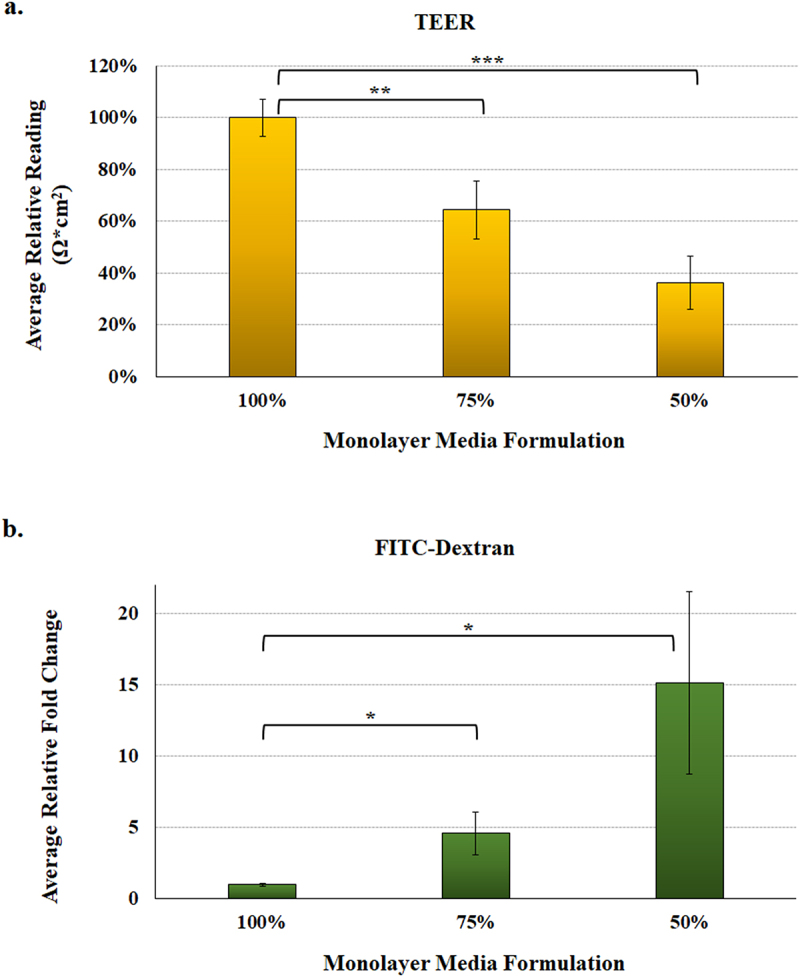
**Malnourished monolayer permeability analysis.** To measure integrity and permeability of the malnourished monolayers, mHIODEM were derived from the D11 and D14 organoid lines, and TEER and diffusion of FITC-dextran were measured. Each measurement occurred following monolayer differentiation with DAPT. Data represent three biologically independent experiments, each with at least two technical replicate wells. For both analyses, the average data ± SEM are plotted. Statistical significance was determined with the Student’s T-test comparing results from the 100% media to the 75% or 50% media. Data were considered significant at a *p* value of < .05 (*, <.05; **, <.01; ***, <.001). Please note the different y-axes for each graph.

**Figure 6.**
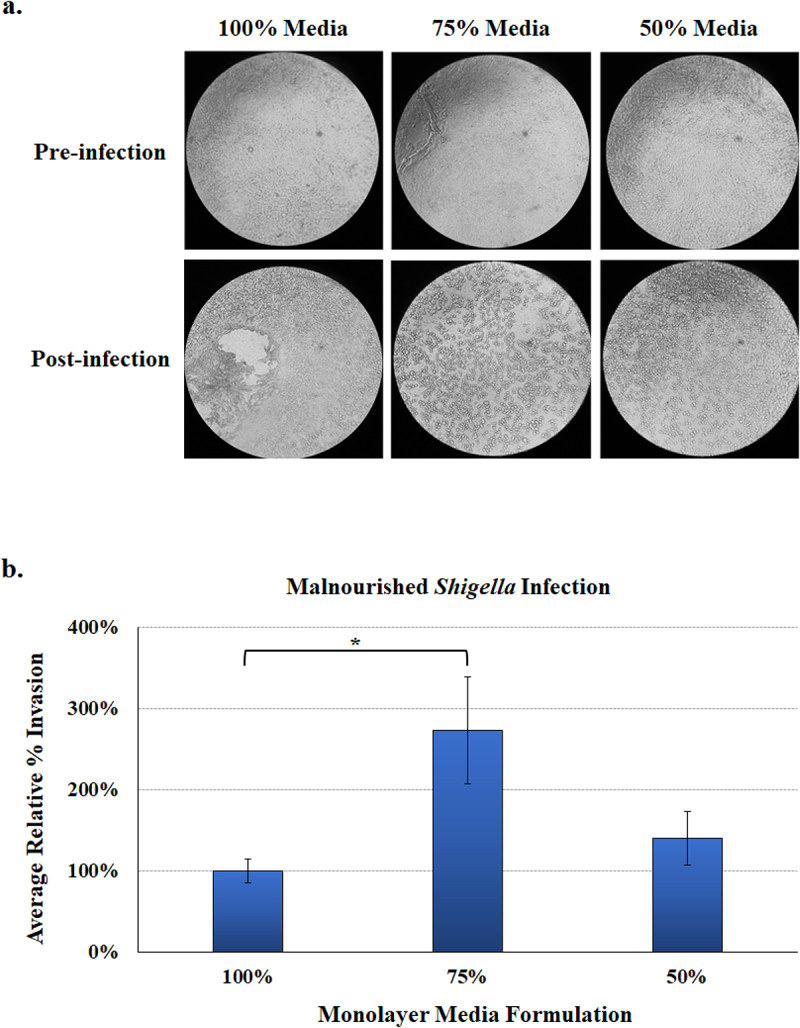
**Malnourished monolayer infection analysis.** Organoids were trypsinized, seeded onto transwells, and cultured in the 100%, 75%, and 50% media formulations. Following 11 days of culturing and a 48-hour differentiation treatment with DAPT, monolayers were subsequently infected with *S. flexneri* strain 2457T. Following apical administration of the bacteria, plates were incubated for 3 hours and then treated with gentamicin to kill the extracellular bacteria.

As noted previously, the human intestinal organoid-derived epithelial monolayer (HIODEM) model offers a reliable and reproducible 2D, human-specific model to examine infection with enteric pathogens.^34^ Thus, we sought to determine if we could establish malnourished monolayers (mHIODEM). After culturing organoids in standard (100%) media for seven days, organoids were trypsinized and seeded onto transwells in the 100%, 75%, and 50% media formulations. Monolayers were cultured and monitored for 11 days, during which transepithelial electrical resistance (TEER) was measured throughout the culturing to assess monolayer integrity.^34,54^ On day 11, all monolayers appeared confluent with TEER readings indicative of an established monolayer^34,55^ (data not shown); and thus, the differentiation reagent DAPT (*N*-[*N*-(3,5-Difluorophenacetyl-L-alanyl)]-S-phenylglycine t-Butyl Ester) was applied to the monolayers for 48 hours to ensure cellular differentiation and mature cells.^34,36^ On day 13, the monolayers were evaluated for barrier integrity (or permeability) and infected with *S. flexneri*. TEER was significantly decreased in the malnourished monolayers in both the 75% and 50% media formulations relative to the 100% control (Figure 5a), indicating reduced barrier integrity and increased transcellular and paracellular permeability of the malnourished monolayers.^56^ Additionally, passage of 4.0 kilodalton fluorescein isothiocyanate (FITC)-dextran molecules is a direct measure of paracellular permeability,^54^ and was significantly increased in both malnourished monolayers, particularly in the 50% media formulation (Figure 5b), confirming the TEER readings and increased permeability of the mHIODEMs. Based on microscopic inspection, all monolayers appeared confluent prior to infection (Figure 6a); and thus, we proceeded with the *S. flexneri* infection analyses. Following apical administration of the bacteria as performed previously,^34^ *S. flexneri* appeared to have a more diffuse infection pattern across the entire monolayer surface in the malnourished conditions compared to a more focused, centralized infection location in the 100% media control (Figure 6a). Cellular invasion rates were induced in the malnourished monolayers, significantly in the 75% media formulation (Figure 6b), indicating that malnourished conditions increase the ability of *S. flexneri* to invade the duodenal epithelium.

**a.** **Transepithelial electrical resistance (TEER)** was evaluated as a measure of resistance multiplied by the area of the transwell (Ω*cm^2^). Data are plotted relative to the 100% media condition, which is set at 100%. There was a significant decrease in TEER for the 75% (**, *p* < .01) and 50% (***, *p* < .001) media formulations, indicating higher transcellular and paracellular permeability under malnourished conditions. There was also a decrease between the 75% and 50% media, but the *p* value was .07.

**b.** **FITC-dextran passage** was evaluated as a measure of paracellular permeability by examining diffusion of the 4 kilodalton FITC-dextran molecule. There was a significant increase in FITC-dextran passage in both the 75% and 50% media formulations (*, *p* < .05), indicating higher paracellular permeability of the malnourished monolayers. There was also an increase in the 50% media relative to the 75% media, but the difference was not significant.

**a.** **Phenotypic analysis of monolayers.** Images were examined at 5X magnifications for pre-infection (top) and post-infection (bottom) monolayers for each media formulation. The images indicate that the monolayers can be established on transwells in the malnourished media conditions. Additionally, the malnourished media enabled a more consistent infection pattern across the monolayer in both the 75% and 50% media formulations, as opposed to a more site-specific infection patter in the 100% media formulation. Images are monolayers derived from the D14 organoid line and are representative of three biologically independent experiments with two technical replicates for each experiment.

**b.** **Bacterial invasion rates** were calculated by dividing the recovery titers (CFU/ml) by the infecting titers and multiplying by 100%. The average results are plotted relative to the 100% media formulation ± SEM. The data represent 4 biologically independent experiments (from organoid lines D14 (3 experiments) and D11 (1 experiment)), and each experiment had at least two replicate wells per condition. The Student’s T-test was used to measure significance, in which there was a significant increase in the invasion rate (*, *p* < .05) in monolayers cultured 75% media formulation relative to the 100% control media.

## Discussion

Experiments using modified culture conditions to study malnutrition are limited, especially with organoids from human donors and using monolayer-based models that enable infection analyses. This methodology provides a novel yet straight-forward approach to deepen the experimental complexity of organoid research. Since research was limited, we decided to use a more macro-level nutrient deprivation approach in which multiple growth factors like sugars, amino acids, and other nutrient components were limited. While we did consider utilizing micro-level deprivation,^57^ *e.g.*, limiting one nutrient such as iron,^58^ zinc,^59,60^ or vitamin A,^61^ we proceeded with macro-level deprivation to better represent the malnourished conditions of LMICs as well as the most updated definition of pediatric malnutrition based on American Society for Parenteral and Enteral Nutrition (ASPEN) guidelines.^62^ The ASPEN workgroup defined pediatric malnutrition as “an imbalance between nutrient requirement and intake, resulting in cumulative deficits of energy, protein, or micronutrients that may negatively affect growth, development, and other relevant outcomes”.^62–64^ Malnutrition is a highly complex physiological state. Our model offers a proof-of-concept approach to study malnutrition as well as a foundation to adapt the model for future studies. A recent study performed robust analyses of duodenal organoids collected from biopsies of EED patients using the organ-on-a-chip technology.^65^ While this study provides important new information on the effects of specific nutrient deprivation on the GI epithelium and included removal of niacinamide and tryptophan from the organoid media, access to both organoids derived from EED patients and the organ-on-a-chip technology is limited. Thus, our malnourished media formulations can be applied and adapted to a wide range of studies, and we look forward to helping researchers advance this approach.

The results of our analyses indicate that we achieved a malnourished state with the 75% and 50% media formulations. The analyses focused on genes that have been implicated in malnutrition, such as cellular differentiation, barrier formation and function, and autophagy.^21–23,66,67^ Additionally, we examined *SPIB* as a marker of M cells, which are critical for the infection cycles of *S. flexneri* and other enteric pathogens like *Salmonella* and *Yersinia*.^34,39,68,69^ Most of the analyses focused on comparison of gene expression patterns, with supporting data from the immunofluorescence and secreted IAP and IL-8 analyses. It is important to note that gene and protein expression can be affected by sampling, whether it is the day of the organoid culture or the time at which biopsy samples are obtained from patients (for the BMI samples). Additional considerations include the length of time and the degree to which malnutrition has been established. We expect that modifications to organoid culturing times or other adjustments will affect gene and/or protein expression levels for targets of interest. Nevertheless, our data demonstrate that the malnourished media formulations enable detection of markers for malnutrition as discussed below. More importantly, the LDH release and the cellular viability analyses confirmed the 75% and 50% media formulations did not cause cytotoxic effects on the organoids, thus confirming this approach can be used to study the effects of malnutrition.

Focusing on cellular differentiation, especially in relation to cell types associated with enteric infection like goblet cells and M cells,^34^ we examined gene expression for the stem cell marker *LGR5* ^46,65^, the lysozyme *LYZ* gene^46,47^, *MUC2* ^38^, and *SPIB* ^34^. Expression of the immature stem cell marker gene *LGR5* was modestly increased in organoids cultured in the 75% and 50% media formulations; and importantly, significantly induced in the low BMI biopsies. Nutrient deprivation typically causes more enterocytes to remain in an immature and undifferentiated state.^46^ Likewise, increased lysozyme produced by Paneth cells is an indication of stem cell crypt expansion since lysozyme is actively involved in keeping LGR5^+^ stem cells in the immature state.^70^ Like *LGR5*, the expression of *LYZ* was modestly increased in the 75% media formulation, but trended toward significantly induced expression in the low BMI samples while the high BMI sample was modestly repressed. While the malnourished organoid culturing times (seven days) could have limited our ability to detect induced stem cell expression of *LGR5* and *LYZ*, the lack of significantly altered expression was likely affected by the induction of mature cells, as indicated by increased *MUC2* and *SPIB* expression and confirmed with the immunofluorescence analysis. The *MUC2* gene encodes mucin-2 that is produced by goblet cells.^38^ We detected significant up-regulation of *MUC2* in both the malnourished organoids and the low BMI biopsy samples. One could expect nutrient deprivation to result in less mucus and goblet cell production, as indicated in animal models and the recent EED organ-on-a-chip analyses.^11,65^ However, a recent study that analyzed tissue directly from patients with EED and undernutrition in Pakistan and Zambia identified increased mucus-producing goblet cells in the duodenal epithelium of this malnourished patient cohort.^71^ In this study the malnourished samples were compared to tissue from patients in the United States with celiac disease, which causes enteropathy without affecting BMIs, suggesting the increased goblet cells could be due to malnutrition. Furthermore, a recent Zambian-based study detected induced goblet cell and mucus gene expression in severely malnourished children relative to stunted children.^72^ Finally, patients with high sugar and high fat diets have been shown to have decreased *MUC2* expression.^73^ We hypothesize that increased mucus production could provide a protective barrier to a permeable, nutrient-deprived epithelium. Meanwhile, the *SPIB* analysis demonstrated the gene was modestly induced in the low BMI biopsies, and significantly induced in the 50% media formulation. The *SPIB* gene is a marker for M cells and induced M cell expression will affect the ability of *S. flexneri* and other enteric pathogens to infect the epithelium.^34,68,69^ Coupled with the induced *MUC2* results and the literature, our data indicate that the malnourished conditions altered cellular differentiation to varying degrees, which has implications for infection patterns of pathogens (see below).

As noted, the barrier integrity of the epithelium is critical to protecting the GI tract from infection, especially from pathogens like *S. flexneri* that invade at the basolateral pole.^35^ Thus, we analyzed both barrier formation and function to evaluate barrier integrity. For barrier formation, the expression of the tight junction genes *OCLN* ^40^ and *CLDN2* ^40–43^ were analyzed.^40,74^ Occludin is associated with epithelial permeability, though the precise mechanism has not been established.^75^ Decreased occludin expression has been reported in undernourished patients,^50^ and the organoids in the 50% media formulation trended toward repression but were not statistically significant. However, the low BMI biopsies had significantly induced *OCLN* expression. Occludin can be increased in some situations to help decrease epithelial permeability,^76^ and these differential results could reflect variations between acute malnutrition of the organoids compared to more chronic malnutrition of the low BMI biopsies. Claudin-2 encodes a pore forming protein in the tight junction complex,^40^ which leads to greater permeability and clinically increased GI fluid loss in the form of diarrhea.^77^ The *CLDN2* gene trended toward induced expression in the organoids cultured in the 75% and 50% media formulations as well as in the low BMI biopsies (Figure 3; although not statistically significant), indicating increased permeability. While there are conflicting data as to whether claudin-2 is induced or repressed in response to undernutrition,^41–43^ convincing evidence supports induced expression.^41,42^ Further, induced claudin-2 is associated with many enteropathies that resemble malnutrition histologically, including Crohn’s disease, ulcerative colitis, HIV enteropathy, celiac disease, and enteric infections, all of which present clinically with diarrhea.^40,43,77^ Regarding barrier function, IAP and IL-8 secretion were examined. IAP is produced by the small intestine, is critical to the antimicrobial and barrier functions of the epithelium, and has been shown to be decreased in humans and rodents deprived of enteral feeding.^48,49^ While the *IAP* gene was not differentially expressed, we detected significantly reduced IAP secretion in the culture supernatants of the malnourished organoids on day seven. The decreased IAP production in our analyses supports the malnourished state, which is decreased after short periods without enteral nutrition and found to be repressed in severely malnourished children.^49,72^ Furthermore, IAP is a major regulator of gut mucosal permeability and plays an important role in gut-microbe interactions,^49^ which is of particular importance in validating our model as IAP is one of the few proteins that has been studied *in vivo* in both humans and rats.^49^ Barrier function was also examined by secretion of IL-8 into the culture supernatants on day seven. While most analyses involve microbial stimulation,^51,52^ we were interested in exploring the basal level of expression in the malnourished state. Both malnourished organoids had reduced IL-8 secretion, which was significantly reduced in supernatants cultured in the 50% media formulation. Interestingly, the siderophore enterobactin, which has a high affinity for iron, can modulate IL-8 expression in the absence of microbial stimulation.^78^ Therefore, it will be interesting to explore how nutrition affects both IL-8 expression and the subsequent vulnerability to enteric pathogens in more detail. Both IAP and IL-8 secretion could be affected by slower growth of the malnourished organoids, particularly with the 50% media; but slower growth could certainly be present in malnourished patients and thus providing important links between malnutrition and enteric infection.

The last gene targets we examined were related to autophagy since undernutrition is known to trigger this critical cellular recycling process during stress and nutrient deprivation.^44,45^ We examined the genes encoding *ATG5* ^45^ and *MTOR*.^44^ The *ATG5* expression trended toward induced expression in the malnourished organoids and low BMI biopsies, while *MTOR* was differentially expressed across treatments. Specifically, *MTOR* was modestly repressed in the 75% media formulation, modestly induced in the 50% media formulation, and significantly induced in both low and high BMI biopsies in which the low BMI samples had the highest induction. It is worth noting that stress and dysfunctional peroxisomes in the GI tract can induce autophagy;^79^ and thus, we cannot rule out that the high BMI sample is not simply responding to stress. Despite the discrepancies, the overall trends indicate that autophagy is induced in the malnourished state. Additionally, since MTOR is the first protein to sense a change in concentration of available amino acids and initiates autophagy,^80^ it is possible that the varying results again reflect an acute malnourished state in the organoids compared to a more chronic malnourished state in low BMI biopsy samples.

Finally, given the importance of enteric pathogens in LMICs and the need to study the effects of malnutrition on infection patterns, we sought to establish malnourished 2D monolayers (mHIODEM) to study infection. This platform also enables better analysis of barrier formation through TEER readings and FITC-dextran passage analyses,^34,54^ which are critical to the assessment of barrier integrity or epithelial cell permeability observed in undernourished intestinal epithelia.^23,81^ We first cultured organoids in standard (100%) media for seven days, then trypsinized the organoids to seed onto transwells and cultured for a total of 13 days in the three media formulations. This culturing duration was two days longer than our standard protocols^34,55^ since the monolayers in the 50% media needed additional time to form due to the slower growth. Nevertheless, the monolayers were successfully established, and we proceeded with evaluation. As noted above, both the TEER and FITC-dextran analyses indicate increased transcellular and paracellular permeability of the monolayers, which confirm the malnourished state of the monolayers. The last step in the proof-of-concept monolayer analyses was to compare infection rates. While *S. flexneri* normally infects the colon due to tissue tropism, the pathogen can infect different segments of the small intestine.^34,52,82^ Furthermore, we were interested in examining if the malnourished duodenal organoids permitted *S. flexneri* to have better access to the basolateral pole for invasion due to both increased barrier permeability and/or M cell expression of the malnourished organoids. *S. flexneri* invaded the malnourished monolayers at higher rates compared to the standard (100%) monolayer, with statistical significance for the 75% media formulation. It is worth noting that the number of bacteria recovered from the 50% media formulation may not reflect the total number of *S. flexneri* that invaded the cells. Host cell lysis during infection reduces the bacterial recovery titers by exposing *S. flexneri* to gentamicin in the media, which is used to kill extracellular bacteria and ensure recovery of intracellular populations.^34^ Further investigation, especially for the 50% media mHIODEMs, is required; however, the results indicate that malnutrition increases *S. flexneri* invasion of duodenal epithelia. The more diffuse infection pattern across the malnourished monolayers, compared to the centralized infection site in the 100% control monolayer, further indicated better access of the epithelium for bacterial invasion due to barrier permeability. Recent advances in technology and more sophisticated methodology have improved our understanding of *S. flexneri* invasion of the epithelium. While the classic basolateral model of invasion following M cell transit in intestinal colonic crypts is still accepted,^35,83^ recent literature has documented improved basolateral access through barrier permeability,^84^ bacterial targeting of goblet cells,^85^ apical surface infection through adherence^86^ and invasion when mechanical flow and peristalsis are considered,^87^ and asymptomatic carriage in malnourished children.^88^ Therefore, future studies comparing these new aspects of *S. flexneri* infection between healthy and malnourished states are very promising to enhance our understanding of pathogenesis. Future analyses will also need to compare infection along the different segments of the small intestine and colon. The malnourished monolayers offer exciting opportunities to understand *S. flexneri* infection in models that represent patients in LMICs.

In all, our data demonstrate that the malnourished media formulations and organoid culturing conditions are feasible and represent important features of malnutrition. This project raises many possibilities for future studies. First, studies of enteric pathogenesis would clearly be a useful application of the model. Assays could examine mechanistic underpinnings of common bacterial and viral pathogens that behave differently in patients with undernutrition or obesity such as *Shigella, Salmonella*, pathogenic *Escherichia coli*, *Vibrio cholerae*, rotavirus, and SARS-CoV2.^5,89–91^ Improved mechanistic understandings of pathogenesis for a global population would ultimately help us develop better vaccines and therapeutics to underserved populations.^7^ Second, the model could also be used to study IBD, specifically in the surgical setting where outcomes are improved with optimized nutritional status. Patients with IBD (Crohn’s disease and ulcerative colitis) often need intestinal resections to manage the disease. Undernutrition at the time of surgery is an independent risk factor for surgical complications.^15^ Thus, this model could be used to target high-impact nutritional supplements that could be provided peri-operatively, possibly reducing morbidity. Further, the model could also be used examine the impact of specific nutrient supplements or depletions on epithelial function and determine if dietary interventions can be used to treat a variety of GI diseases, including EED. Finally, as techniques advance to accurately incorporate microbiota analyses with organoids, comparisons between healthy and under- or over-nourished models can further dissect the complex relationship of the microbiota with the mucosal environment, as well as the bidirectional cross-talk of the microbiome and epithelium.^92^ It is becoming clear that certain intrinsic characteristics and functions of the epithelium, which can be modified by environmental exposures such as diet, toxins, and medications, lead to changes in the epithelium that ultimately impact the mucosal interaction with and selection of microbes within an individual’s microbiome. As microbiome-related studies are being conducted with great interest, the model we have developed lends itself to enhance these analyses.^92,93^ In all, there are many useful and exciting uses for a relatively accessible and easily reproducible malnourished organoid model in biomedical research.

## Materials and methods

### Organoids and cell culture conditions

The protocol has been adapted from previous publications.^34,36,55^ Briefly, stem cells derived from donor duodenal biopsies were maintained in Matrigel culture in a 1:1 mixture of ISC and L-WRN conditioned media containing the inhibitors Y-27632 and A-83-01. Cells were seeded at a density of 10,000 cells per Matrigel dome and grown for seven days in culture. We set up three lines of duodenal organoids from three different healthy patient donor lines (referred to as D11, D14, and D15), and plated each donor line in wells with three different concentrations of media (100%, 75%, and 50%, see below) for seven days. All human sample collection was approved by Institutional Review Board (IRB) protocol 2014P001908 of the Massachusetts General Hospital, Boston, MA. Donor tissue was obtained from consenting patients undergoing medically required endoscopies, as determined by a licensed physician. All subjects provided written informed consent for samples to be used for research purposes.

The three D11, D14, and D14 organoid lines were cultured in three different media concentrations, two of which had reduced concentrations of growth factors, carbohydrates, and proteins to generate the 75% and 50% media formulations relative to the 100% control media formulation (Figure 1). This process was intended to simulate the epithelium from globally malnourished individuals in which several nutrient deficiencies have been established, including extensive carbohydrate and protein deficiencies.^2,7,40^ Organoid media is comprised of 50% L-WRN and 50% ISC media, and nutrient reductions were performed by substituting components of the nutrient-rich ISC media with a solution of 1X PBS containing the same total amount of N2, B27, insulin, N-acetylcysteine, epidermal growth factor (EGF), and gastrin. In the test conditions, either half (75%) or all (50%) of the ISC media was removed and replaced with the enhanced PBS solution. This approach predominately reduced the overall concentrations of proteins, carbohydrates, lipids, and vitamins in the media since the ISC media is composed predominately of FBS and DMEM/F12. The L-WRN is conditioned media supplement based on DMEM/F-12 and the growth factors Wnt3a, R-spondin 3 and Noggin, which are essential for organoid growth.^36^ Organoids were plated in 6-well plates at a density of approximately 1 × 10^6^ cells/well in 1 ml media, and media were replenished on days three and five. On day seven, cells and media were collected and flash frozen at −80°C until further use. This process was repeated three times for each D11, D14, and D15 sample in each media formulation (*i.e.*, three biological replicates for each D11, D14, and D15 sample in the 100%, 75%, and 50% media). To determine if significant cellular lysis occurred during the course of the seven days of culture, the lactate dehydrogenase (LDH) assay was measured via the Promega Cytotoxicity Assay (Promega, Madison, WI) according to manufacturer’s instructions with a positive cell lysis control.^34^ Finally, to compare cellular counts and confirm cellular viability, D14 organoids were trypsinized on day seven,^34^ stained with trypan blue, and examined on a Countess Automated Cell Counter (Thermo) according to manufacturer’s instructions.

### Control duodenal biopsies

As a patient-derived control for the organoid analyses, frozen duodenal tissue biopsy samples from pediatric patients (three low BMI samples with three age and sex matched healthy BMIs controls, and one very high BMI sample) from a pediatric biorepository were obtained. All human sample collection was approved by Institutional Review Board (IRB) protocol 2016P000949 of the Massachusetts General Hospital, Boston, MA. Donor tissue was obtained from consenting patients undergoing medically required endoscopies, as determined by a licensed physician. All subjects provided written informed consent for samples to be used for research purposes. The undernourished patients had weight-for-height ratios (Z scores)/BMIs at or just above −2, thereby putting the donors in the category of mild to moderate malnutrition.^62^ The healthy control patients were age and sex matched to the undernourished patients and had Z scores/BMIs between the 25^th^ and 75^th^ percentile. The high BMI biopsy sample was from a patient with a BMI in the morbid obesity range. RNA from all frozen biopsy samples was extracted and qPCR was performed for gene expression (see the quantitative RT-PCR below for method).

### Duodenal organoid monolayer cultures

Monolayers from the organoids were generated as previously described to produce an epithelial barrier on a 2D surface with apical and basolateral sides.^24,34^ This platform enables analysis of TEER and passage of FITC-dextran molecules for assessment of barrier integrity and epithelial barrier function.^34,54^ Organoids were cultured in Matrigel domes in 100% standard media until day seven as described above. To establish monolayers, cultured organoids were trypsinized into single cells and seeded at 1 × 10^5^ cells/well onto uncoated polyester transwell membranes with a 0.4 μm pore size (24 well-plate, #3470, Corning) as previously described.^34^ Monolayers were cultured in the varying 100%, 75%, and 50% media formulations, which were freshly supplemented with the Y-27623 inhibitor and changed every other day. TEER measurements and microscopic observation were used to monitor confluence. On day 11, the transwells appeared confluent regardless of media concentration. To promote cell differentiation and maturation, confluent monolayers were apically treated with 5 μM DAPT (Calbiochem) for 48 hours, whereas basolateral media contained only the different formulations of the L-WRN/ISC media.

### Quantitative reverse transcription polymerase chain reaction (RT-PCR) of malnourished organoid cultures

RNA was extracted from organoids cultured in the three different media formulations frozen on day seven, as well from the duodenal biopsy tissue, as previously described.^34^ Briefly, RNA was extracted using Trizol (ThermoFisher) and the Direct-Zol (Zymo) RNA extraction kit. RNA was treated with on-column DNase with Turbo DNase (Ambion). Subsequently, RNA was converted to cDNA using Thermo Scientific Maxima First Strand cDNA Synthesis Kit, and gene expression was quantified by the SYBR Green (Perfecta) method using the CFX96 real-time PCR detection system (Qiagen, Venio, NL). The relative threshold cycle (ΔΔCT) method was used for determining gene expression levels across samples relative to the 18S housekeeping reference gene as previously described.^34^ The following genes were analyzed: mucin-2 (*MUC2*) for goblet cells;^38^ *SPIB* for M cells;^34^ occludin (*OCLN*)^40,74^ and claudin-2 (*CLDN2*)^40–43^ for barrier formation, *LGR5* for intestinal stem cells,^34,46^ lysozyme (*LYZ*) for Paneth cells,^46,47^ *ATG5* and *mTOR* for autophagy,^44,45^ and intestinal alkaline phosphatase (*IAP*).^48^ Please refer to Table 1 for primer sequences.

**Table 1.** Primers used in this study.

Gene	Forward Primer	Reverse Primer
*18S*	5’-AGAAACGGCTACCACATCCA-3’	5’-CCCTCCAATGGATCCTCGTT-3’
*MUC2*	5’-GCCAGCTCATCAAGGACAG-3’	5’-GCAGGCATCGTAGTAGTGCTG-3’
*SPIB*	5’-GGCCACACTTCAGCTGTCT-3’	5’- CCCCTGTAGCCATCCATTCC-3’
*OCLN*	5’-ACAAGCGGTTTTATCCAGAGTC-3’	5’-GTCATCCACAGGCGAAGTTAAT-3’
*CLDN2*	5’-ACCTGCTACCGCCACTCTGT-3’	5’-CTCCCTGGCCTGCATTATCTC-3’
*MTOR*	5’-ATGCTTGGAACCGGACCTG-3’	5’-TCTTGACTCATCTCTCGGAGTT-3’
*ATG5*	5’-AAAGATGTGCTTCGAGATGTGT-3’	5’-CACTTTGTCAGTTACCAACGTCA-3’
*LYZ*	5’-CTTGTCCTCCTTTCTGTTACGG-3’	5’-CCCCTGTAGCCATCCATTCC-3’
*IAP*	5’-CTTTGGTGGCTACACCTTGC-3’	5’-CTCGCTCTCATTCACGTCTGG-3’
*LGR5*	5’-CTCCCAGGTCTGGTGTGTTG-3’	5’-GAGGTCTAGGTAGGAGGTGAAG-3’

The 5’ to 3’ sequence of each primer pair (forward and reverse) are provided for each gene target.

### Protein expression analyses in malnourished organoids

Immunofluorescence analysis was performed on D14 organoids cultured to day seven. Organoids were recovered from Matrigel in 5 mM EDTA containing PBS for one hour on ice, fixed with paraformaldehyde, and paraffin embedded according to the Stemcell Technologies protocol (https://www.stemcell.com/technical-resources/performing-icc-staining-epithelial-organoids.html#part-6) and as previously described.^94^ The organoids were then processed for sectioning and unstained slide mounting at the Histopathology Research Core at MGB. Subsequently, the sections were deparaffinized, rehydrated, and immunostained following antigen retrieval and cell permeabilization and blocking according to the Stemcell Technologies protocol. Primary antibodies were used at the following concentrations in 1% bovine serum albumin in PBS: mouse anti-ZO-1 (Invitrogen 339100) at a dilution 1:200, rabbit anti-SpiB (Cell Signaling Technologies 14323S (D3C5E) at a dilution 1:200, and rabbit anti-human-Muc2 (Santa Cruz sc15334 (H300) at a dilution 1:200. Secondary antibodies used were goat anti-mouse Alexa Fluor 594 (Invitrogen A32742) at 8 µg/mL in PBS, and goat anti-rabbit Alex Fluor 488 (Invitrogen R37116) by diluting 1 drop in 500 µl of PBS per manufacturer’s instructions. DNA was stained with Hoechst 33342 trihydrochloride, trihydrate (Invitrogen H3570) and used at 5 µg/ml. Confocal immunofluorescence was performed with a Nikon Eclipse microscope with a 60X objective.

To evaluate IAP and IL-8 secretion, organoid culture supernatants were collected from cultures of D11, D14, and D15 on day seven. The analyses used supernatants from three different experiments (biological replicates) in triplicate (technical replicates) for the 100%, 50% and 75% media formulations, which were stored at −80°C until analysis. IAP secretion was assessed via the alkaline phosphatase assay kit (Colorimetric; ab83369; ABCAM) according to manufacturer’s instructions. Media controls were incubated for the same amount of time and conditions in plate wells lacking cells, and these values were subtracted from the results to control for media color affecting the results of the assay. IL-8, a pro-inflammatory chemokine produced by intestinal epithelium, was conducted using R&D Human CXCL8/IL-8 DuoSet ELISA per manufacturer’s instructions as previously described.^34^ Supernatants were uniformly diluted for the analyses due to high concentrations in the samples.

### Malnourished monolayer analyses

Once the malnourished monolayers (mHIODEM) were established, three analyses were used to determine barrier integrity and susceptibility to infection. Analyses took place on day 13 of culture, which was 48 hours after the addition of DAPT for cellular differentiation. First, TEER was used to examine transcellular and paracellular permeability,^56^ and monitored using a TEER apparatus (World Precision Instruments, Sarasota, FL) per manufacturer’s instructions as previously described.^34^ TEER readings are reported prior to media changes and/or FITC-dextran passage analysis. Second, paracellular permeability^54^ was determined by measuring the flux of FITC-dextran (molecular weight of 4.0 kilodaltons, Sigma-Aldrich) from the apical transwell chamber, through the differentiated monolayers, and into the basolateral chamber of each well. For the analysis, the media was changed with 1:1 media of the appropriate 100%, 75%, or 50% formulations on the basolateral side, and all the wells were treated with a standardized volume and concentration of FITC-dextran in the 50% media formulation on the apical side after two hours of incubation as previously described.^54^ Results were read on a BioTek fluorescence plate reader at 488 nm. Finally, to determine the effect of malnourishment on infection, malnourished monolayers were infected with *S. flexneri* strain 2457T as previously described.^34^ Briefly, bacteria were grown overnight in TSB media at 37°C, subcultured the following day to an OD_600_ of 0.7 for mid-log phase, washed 3 times with 1X PBS, resuspended in DMEM/F12 media, and placed on the apical surface of the monolayers in 40 µls. Infecting titers were determined by plating serial dilutions of the inoculums on tryptic soy agar plates supplemented with the dye Congo red (CR plates) to differentiate between virulent and avirulent colonies.^34^ After 3 hours of incubation, monolayers were washed 3 times with 1X PBS and resuspended in DMEM/F12 media with 50 µg/ml gentamicin to kill the extracellular bacteria. After 3 hours of incubation, monolayers were washed 3 times with 1X PBS and lysed with pre-warmed 1% Triton-x-100. To determine the number of intracellular bacteria, monolayer lysates were dilution plated onto CR plates, and the percent recoveries were determined by dividing the recovery titers in colony forming units per ml (CFU/ml) by the infecting titers in CFU/ml and multiplying by 100%.

### Statistical analyses

All analyses were performed with biological and technical replicates. Please refer to each figure legend for details for each assay. To determine statistical significance, *p* values were calculated using the Student’s T-test to compare each malnourished formulation to the 100% control, or to compare the 75% formulation to the 50% formulation. All *p* values < 0.05 were considered significant. Analyses plot the average results ± the standard error of the mean (SEM). Data plotted relative to the 100% media formulation are noted.

## Data Availability

The authors confirm that the data supporting the findings of this study are available within the article.
